# The Editor's Letter-Box

**Published:** 1887-10-01

**Authors:** M. Chalk


### The Editor's Letter-Box.

[Our Correspondents are reminded that prolixity is a great bar to publication, and that brevity of style and conciseness of statement greatly facilitate early publication.]

Can you or any of your readers give me any information respecting an institution or a home where an elderly lady could be received who is afflicted as stated below ?

10, Lawford Road, Camden Road. M. Chalk.

A lady, seventy-seven years of age, afflicted with chronic rheumatism, is in the possession of a small income amounting to about seven shillings per week, which she would be willing to pay towards her maintenance ; beyond that she is destitute, and has no relatives capable of rendering her further assistance. M. C.

